# Ozone Improves the Aromatic Fingerprint of White Grapes

**DOI:** 10.1038/s41598-017-16529-5

**Published:** 2017-11-24

**Authors:** Susana Río Segade, Mar Vilanova, Simone Giacosa, Irene Perrone, Walter Chitarra, Matteo Pollon, Fabrizio Torchio, Paolo Boccacci, Giorgio Gambino, Vincenzo Gerbi, Luca Rolle

**Affiliations:** 1 Università degli Studi di Torino, Dipartimento di Scienze Agrarie, Forestali e Alimentari, Largo Paolo Braccini 2, 10095 Grugliasco, TO Italy; 2 Misión Biológica de Galicia, Spanish National Research Council (CSIC), El Palacio-Salcedo, 36143 Pontevedra, Spain; 3 Consiglio Nazionale delle Ricerche, Istituto per la Protezione Sostenibile delle Piante, Strada delle Cacce 73, 10135 Torino, Italy; 4 Present Address: Università Cattolica del Sacro Cuore, Istituto di Enologia e Ingegneria Agro-Alimentare, Via Emilia Parmense 84, 29122, Piacenza, Italy; 5 Present Address: Centro di Ricerca per la Viticoltura e l’Enologia - Consiglio per la Ricerca in Agricoltura e l’Analisi dell’Economia Agraria, Via XXVIII Aprile, 31015, Conegliano, TV Italy

## Abstract

Ozone, a powerful oxidative stressor, has been recently used in wine industry as sanitizing agent to reduce spoilage microflora on grapes. In this study, we evaluated ozone-induced metabolic and molecular responses during postharvest grape dehydration. Ozone increased the contents of total volatile organic compounds (VOCs), which have a great impact on the organoleptic properties of grapes and wines. Among terpenes, responsible for floral and fruity aroma, linalool, geraniol and nerol were the major aromatic markers of Moscato bianco grapes. They were significantly affected by the long-term ozone treatment, increasing their concentration in the last phases of dehydration (>20% weight loss). At molecular level, our results demonstrated that both postharvest dehydration and ozone exposure induce the biosynthesis of monoterpenes via methylerythritol phosphate (MEP) pathway and of aldehydes from lipoxygenase-hydroperoxide lyase (LOX-HPL) pathway. Therefore, transcriptional changes occurred and promoted the over-production of many important volatile compounds for the quality of white grapes.

## Introduction

In grapevine (*Vitis vinifera* L.), several classes of secondary metabolites are synthetized in berries, which determine the characteristics and the quality of each cultivar and of the wines produced. Among these metabolites, many volatile organic compounds (VOCs) are present in grape berries and some of them play a key role in the aromatic quality of white wines^1^. From the sensory point of view, aroma is one of the most appreciated features in assessing wine quality.

Monoterpenes are grape-derived active odorants, which are responsible for the floral and citrus attributes of wines, particularly in several aromatic varieties^2,3^. Odorless glycosylated monoterpenes can be also present in grape berries and the free forms can be released by acid or enzyme hydrolysis during wine making and aging, contributing these precursors to enhance the wine aroma^1^. The predominant monoterpenes in white wines are linalool, geraniol, nerol, α-terpineol, β-citronellol, ho-trienol, and limonene^4^. Monoterpenes are also considered health-promoting compounds because of anticancer, cardiovascular protective, anti-inflammatory, antioxidative, antimicrobial, and antiviral properties^5^. In grapevine berries, the biosynthesis of monoterpenes occurs through the methylerythritol phosphate (MEP) pathway starting with the condensation of glyceraldehyde 3-phosphate and pyruvate in plastids^6^. The biosynthesis increases after véraison and in the latest stages of development before harvest^7^.

In addition to monoterpenes, C_6_-compounds (aldehydes, alcohols, and esters) are another class of VOCs particularly abundant in grapevine berries and characterized by an evocative ‘green’ aroma of fresh cut leaves (Green Leaf Volatiles)^8^. C_6_-compounds are produced by oxidative cleavage of polyunsaturated fatty acids (PUFAs), in particular linoleic and linolenic acids. This process occurs through the lipoxygenase–hydroperoxide lyase (LOX–HPL) pathway, in which lipoxygenase (LOX), hydroperoxide lyase (HPL) and alcohol dehydrogenases (ADHs) are the major enzymes involved^9–12^.

High alcohols and aldehydes are usually produced during the fermentation process by yeasts and bacteria in relation to sugar and amino acid metabolism^13^. However, many of these compounds can be produced by plants^14^.

In plants, VOCs are generally influenced by many stresses (e.g. wounding, drought, air pollution, heat, flooding) that can modify their constitutive emissions and elicit the production of novel compounds (induced emissions)^15^. An increased emission of VOCs, such as C_6_ compounds, isoprene, and monoterpenes, was observed in photosynthetic tissues in response to abiotic stresses^16–19^, suggesting an involvement of these compounds in enhancing plant tolerance^20^ and in a general defense priming signal in nature^21^. Among the abiotic stressors expected to increase in the global change scenario, the ozone represents a strong oxidizing agent inducing several physiological and metabolic changes^22^. In particular, ozone induces in cells the production of reactive oxygen species (ROS), such as hydrogen peroxide (H_2_O_2_)^23^. The plants react to the excess of ROS inducing several antioxidant enzymes (e.g. catalase, ascorbate peroxidase, glutathione peroxidase), which work in concert against the ROS accumulation^24^. Interestingly, it has been reported that volatile isoprenoids can mitigate the effects of oxidative stress, including the one caused by ROS production in plant cells^20^.

Ozone, since its recognition as a safe food disinfectant, has been mostly used in postharvest fruits and vegetables against indigenous microbiota. In wine industry, several applications have been proposed at different stages in winemaking to reduce spoilage microorganisms in grapes, barrels, and tanks^25–28^. Postharvest ozone treatment of grape berries would make it possible to reduce the use of sulfur dioxide (SO_2_) in winemaking and thereby to minimize the deleterious effects of SO_2_ on wine aroma quality and human health. In grapevine, some authors have reported changes in secondary metabolites of grapes after postharvest ozone fumigation because of defensive biochemical, genetic or transcriptional mechanisms. Most of the information available deals with the positive effect of postharvest ozone treatments on the accumulation of phenolic compounds. Artés-Hernández *et al*.^29^ observed an increase of resveratrol for Napoleon ozone-treated table grapes. The ozone treatment overnight of Petit Verdot grapes increased anthocyanin content^30^. Total phenols and hydroxycinnamic acids of Sauvignon blanc grapes increased after 8 and 16 h ozone treatment^31^. During postharvest dehydration, the shock ozone treatment of Pignola grapes preserved the total contents of polyphenols and anthocyanins^32^.

Considering the defensive response of grapevine berries to environmental stresses, it can be hypothesized that the application of a postharvest “controlled” stress could increase their aromatic potential. The postharvest dehydration of grapevine berries is a process that is gaining great interest in the last years for the production of special wines. This process represents a good chance to study the compositional and metabolic effects of long-term ozone treatment on grape VOCs. Postharvest dehydration itself induces metabolic changes affecting berry compositional parameters to a different extent depending on the grapevine genotype and withering environmental conditions^33^. In aromatic varieties, very few studies evaluated the impact of the dehydration process on the volatile profile of grape berries and/or wines^34^.

Despite the importance of VOCs in white wine grapes, little is known about the effect of long-term ozone treatment and/or dehydration process particularly in aromatic cultivars. In this work, we have studied the influence of postharvest dehydration in ozone enriched atmosphere on the volatile composition in Moscato bianco aromatic white cultivar (*Vitis vinifera*). Changes in the accumulation of free and glycosidically-bound volatile compounds, and in the molecular pathways involved in the biosynthesis of C_6_-compounds and monoterpenes were assessed at different stages of grape dehydration under controlled environmental conditions.

## Results

### Ozone effects on chemical parameters

Changes in chemical parameters of Moscato bianco grapes were found at different stages of postharvest dehydration in ozone-enriched atmosphere (OZ) *versus* air (AR) (Table 1). A significant effect of ozone treatment was observed on glucose, fructose and glycerol when berries were dehydrated until reaching 10 and 20% of weight loss (P10 and P20, respectively), increasing their contents in relation to air-treated grapes. However, the contents of glucose, fructose and glycerol were significantly lower for ozone-treated grapes at a dehydration of 30% (P30). As dehydration progressed, the contents of glucose and fructose increased for both air and ozone treatments. With the exception of P20, the air-treated samples also showed an increasing accumulation of glycerol, reaching the maximum content at P30. However, the trend was not evident for ozone-treated grapes, for which glycerol decreased at P15 and P30.

**Table 1:** Chemical composition of Moscato bianco grapes treated with ozone (OZ) and air (AR) during postharvest dehydration.

**Chemical parameters**	**P0**	**P5-AR**	**P5-OZ**	**Sig**.	**P10-AR**	**P10-OZ**	**Sig**.	**P15-AR**	**P15-OZ**	**Sig**.	**P20-AR**	**P20-OZ**	**Sig**.	**P30-AR**	**P30-OZ**	**Sig**.
Weight loss	0	5%			10%			15%			20%			30%		
Glucose (g/L)	137.2	156.2	150.8	*	165.8	175.2	**	179.0	181.1	ns	182.0	205.8	***	234.8	208.8	***
Fructose (g/L)	146.2	168.3	164.4	ns	181.2	190.7	**	195.2	199.8	ns	202.0	222.4	***	253.8	232.5	***
Titratable acidity (g/L)	5.5	4.9	5.0	ns	4.8	4.0	***	3.6	4.9	***	4.1	4.0	ns	4.1	4.1	ns
pH	3.43	3.52	3.45	ns	3.61	3.68	ns	3.83	3.65	**	3.66	3.84	**	3.95	3.72	***
Tartaric acid (g/L)	7.14	7.88	7.56	ns	6.36	6.70	***	5.93	7.45	ns	5.65	5.27	ns	6.19	5.76	ns
Malic acid (g/L)	1.80	1.22	1.21	ns	1.87	1.54	*	1.19	1.47	*	1.53	1.79	ns	1.91	1.74	ns
Citric acid (g/L)	0.18	0.18	0.17	ns	0.28	0.33	ns	0.40	0.26	**	0.34	0.34	ns	0.52	0.43	*
Glycerol (g/L)	0.00	0.19	0.12	ns	0.69	1.33	**	1.61	0.73	***	0.73	2.58	***	3.69	0.96	***

Asterisks denote significant differences between treatments at the same stage of dehydration according to Student’s t-test (P < 0.05, n = 3): *^, ^**and *** indicate significance at *p* < 0.05, *p* < 0.01 and *p* < 0.001, respectively; ns indicates no significant difference. Titratable acidity expressed in g/L as tartaric acid. P: sampling point at a defined weight loss (0, 5, 10, 15, 20, and 30%).

Regarding acids, titratable acidity decreased significantly in grape berries dehydrated in ozone enriched atmosphere at P10 but increased in those dehydrated at P15 when compared with air atmosphere. These differences are directly related with the content of malic acid.

Tartaric acid showed an increased content in ozone-treated grapes *versus* air at P10. However, a significant decrease was observed in the content of citric acid when grapes were dehydrated at P15 and P30 in the presence of ozone. During dehydration, the trend depended on each acid, tartaric acid contents decreased in grapes dehydrated under air and ozone-enriched atmosphere but citric acid increased reaching the maximum content at P30. No evident trend was observed for malic acid.

### Ozone effects on total VOCs

**Figure 1:**
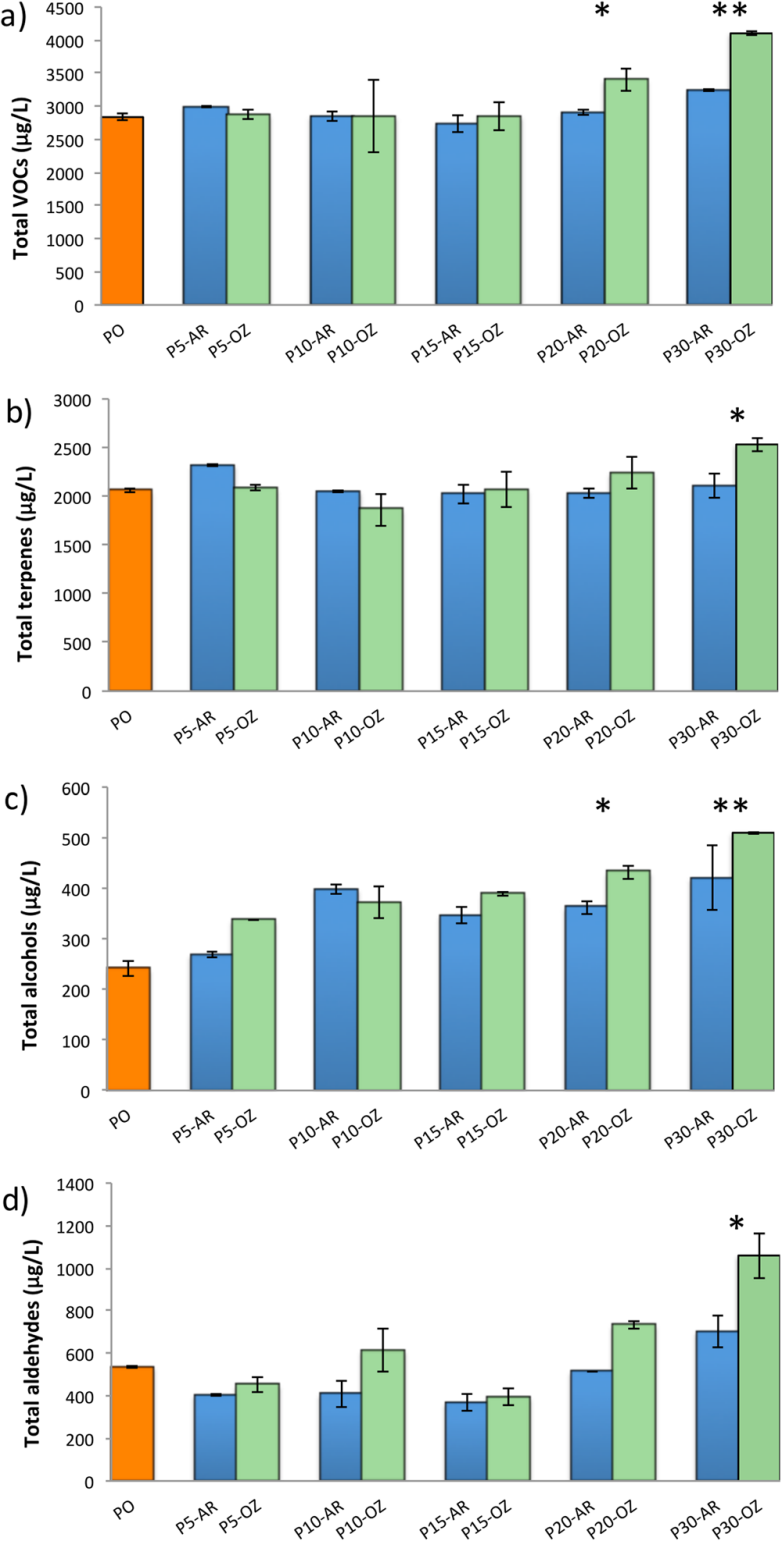
Total volatile composition of Moscato bianco grapes during dehydration. The figure shows the total content of volatile compounds (**a**) and of the different volatile families including terpenes (**b**), alcohols (**c**), and aldehydes (**d**) at different dehydration levels in ozone enriched atmosphere (OZ) and air (AR). Error bars correspond to standard deviations. Asterisks denote significant differences between treatments at the same stage of dehydration according to Student’s t-test (P < 0.05, n = 3): * and ** indicate significance at *p* < 0.05 and *p* < 0.01, respectively. P0, P5, P10, P15, P20, and P30 indicate sampling point at a defined weight loss of 0, 5, 10, 15, 20, and 30%, respectively.

The changes in total contents of volatile compounds, terpenes, alcohols and aldehydes for Moscato bianco grapes at different levels of dehydration in ozone enriched atmosphere *versus* air (control) are shown in Fig. 1. At P20 and P30, a significantly higher content of total volatile compounds was observed in ozone-treated samples. This increase at P30 was due to the higher contents of terpenes, alcohols, and aldehydes, whereas the berries dehydrated at P20 in ozone enriched atmosphere were significantly richer only in alcohols. It is important to take into account that the alcohols and aldehydes were more abundant in ozone-treated berries from the first stages of the postharvest dehydration process, but this trend was not observed for terpenes until P20. In fact, total alcohols exhibited a significantly higher content at P5 in ozone enriched atmosphere in relation to control samples.

The free and bound fractions of VOCs showed different behavior when ozone was applied during dehydration of Moscato bianco grapes, as detailed below.

### Free volatile compounds

Table 2 shows the content of free volatile compounds found in Moscato bianco grapes at different stages of dehydration in ozone enriched atmosphere *versus* air. A total of 33 volatile compounds were identified and quantified (18 terpenes, 9 alcohols, and 6 aldehydes).

**Table 2:** Free volatile composition of Moscato bianco grapes treated with ozone (OZ) and air (AR) during postharvest dehydration.

**Free compounds (µg/L)**	**PO**	**P5-AR**	**P5-OZ**	Sig.	**P10-AR**	**P10-OZ**	Sig.	**P15-AR**	**P15-OZ**	Sig.	**P20-AR**	**P20-OZ**	Sig.	**P30-AR**	**P30-OZ**	Sig.
D-Limonene	2.88	2.76	0.16	**	0.05	0.09	ns	0.84	0.71	ns	0.03	2.25	**	0.87	1.83	ns
β-Phellandrene	1.92	1.84	0.18	***	0.20	0.66	ns	0.33	0.19	ns	0.24	0.44	ns	0.71	0.93	ns
*(E)*-Ocimene	2.54	2.24	1.34	ns	1.26	0.67	ns	0.67	0.54	ns	0.81	1.36	ns	0.19	1.66	**
*(Z)*-Ocimene	3.08	2.84	1.51	***	1.52	1.07	*	0.85	0.69	ns	1.30	1.51	ns	1.23	1.80	**
(+)-4-Carene	0.76	0.88	0.43	*	0.37	0.28	ns	0.22	0.20	ns	0.74	0.45	ns	0.48	0.42	ns
*trans*-Rose oxide	5.97	5.22	2.30	***	2.28	1.52	ns	1.22	1.08	ns	1.81	2.51	ns	3.16	2.14	ns
*cis*-Rose oxide	1.09	1.08	0.32	***	0.29	0.26	ns	0.15	0.17	ns	0.35	0.53	ns	0.77	0.51	ns
*trans*-Furan linalool oxide	0.83	0.81	0.19	*	0.18	0.23	ns	0.18	0.06	ns	0.08	0.31	ns	0.72	0.56	ns
*cis*-Furan linalool oxide	1.18	1.29	0.05	***	0.51	0.17	*	0.13	0.41	ns	0.21	0.91	***	0.72	1.01	ns
Linalool	205.51	165.91	83.78	***	53.87	40.65	ns	22.29	35.56	ns	34.84	30.53	ns	18.17	36.81	**
Ho-trienol	0.71	0.67	0.11	***	0.15	0.15	ns	0.03	0.07	ns	0.08	0.29	**	0.27	0.34	ns
Geranial	0.38	0.53	0.31	ns	0.19	0.22	ns	0.20	0.12	ns	0.27	0.43	ns	0.28	0.39	ns
α-Terpineol	1.23	1.39	0.28	***	0.66	0.44	ns	0.28	0.33	ns	0.34	0.63	ns	0.58	0.67	ns
Neral	1.09	1.56	0.80	*	0.52	0.22	ns	0.40	0.43	ns	0.31	1.30	*	0.89	1.33	ns
*trans*-Pyran linalool oxide	2.06	2.20	0.87	***	0.71	0.53	ns	0.17	0.41	ns	0.71	1.09	ns	1.20	1.53	ns
Citronellol	2.50	3.06	2.03	ns	2.96	1.96	ns	1.50	0.88	ns	2.74	3.43	ns	2.65	3.72	ns
Nerol	11.28	15.17	9.29	*	8.32	6.51	ns	5.01	4.15	ns	9.90	17.01	*	4.63	20.96	***
Geraniol	23.18	23.95	16.35	*	17.34	12.49	ns	9.23	8.39	ns	19.62	32.97	***	14.47	37.83	***
*Total free terpenes*	268.20	233.41	120.29	***	91.39	68.11	ns	43.70	54.40	ns	74.36	97.95	ns	51.98	114.43	***
Pentanol	0.84	1.39	1.07	ns	0.79	0.73	ns	0.89	0.79	ns	0.93	1.57	ns	1.34	1.49	ns
3-Methyl-2-buten-1-ol	0.20	0.72	0.24	ns	0.29	0.15	ns	0.35	0.25	ns	0.28	0.52	ns	0.17	0.55	ns
Hexanol	60.63	57.92	31.34	**	61.48	47.09	ns	36.95	40.33	ns	62.06	85.76	ns	71.48	113.25	**
*(E)*-3-Hexen-1-ol	6.57	4.20	1.60	**	4.62	3.34	ns	2.25	2.78	ns	3.01	4.64	*	3.42	4.06	ns
*(Z)*-3-Hexen-1-ol	36.34	24.15	10.86	ns	40.00	11.96	**	17.01	26.39	ns	26.81	43.76	*	37.76	74.28	***
2-Ethylhexanol	4.78	2.53	4.29	ns	4.34	3.55	ns	1.42	2.36	ns	2.35	3.51	ns	3.83	2.87	ns
Octanol	0.78	0.80	0.63	ns	0.37	0.36	ns	0.54	0.25	ns	0.51	1.03	*	0.22	1.07	**
Benzyl alcohol	0.83	1.10	1.25	ns	1.70	0.40	*	0.64	0.40	ns	0.29	1.61	*	0.53	1.77	ns
2-Phenylethanol	1.47	2.57	2.64	ns	2.44	2.27	ns	0.66	1.76	ns	1.34	2.03	ns	1.99	2.43	ns
*Total free alcohols*	112.44	95.37	53.91	ns	116.03	69.85	*	60.69	75.31	ns	97.59	144.43	*	120.73	201.78	***
Hexanal	345.41	280.93	306.29	ns	264.52	403.98	ns	257.76	271.86	ns	334.07	506.45	ns	419.77	652.27	*
Heptanal	2.36	1.82	1.97	ns	0.87	1.10	ns	1.35	0.33	ns	0.94	2.78	**	1.57	1.60	ns
Octanal	0.48	0.20	0.70	*	0.37	0.12	ns	0.21	0.06	ns	0.44	0.75	ns	0.35	0.39	ns
Nonanal	1.23	2.25	2.74	ns	1.81	2.22	ns	1.25	1.26	ns	0.69	3.35	**	2.28	1.48	ns
Benzaldehyde	1.64	1.81	3.93	ns	2.03	7.25	**	0.76	0.84	ns	0.97	1.55	ns	11.17	4.00	**
*(E)*-2-Hexenal	175.05	108.17	121.57	ns	123.94	184.70	ns	93.84	105.00	ns	163.82	205.80	ns	256.38	384.51	**
*Total free aldehydes*	526.18	395.18	437.21	ns	393.55	599.37	ns	355.18	379.35	ns	500.94	720.66	ns	691.51	1044.24	*

Asterisks denote significant differences between treatments at the same stage of dehydration according to Student’s t-test (P < 0.05, n = 3): *^, ^**and *** indicate significance at *p* < 0.05, *p* < 0.01 and *p* < 0.001, respectively; ns indicates no significant difference. P: sampling point at a defined weight loss (0, 5, 10, 15, 20, and 30%).

Total free terpenes showed a decreasing trend during dehydration for both treatments (air and ozone), although the minimum content was found at P15. However, total free alcohols and aldehydes showed an opposite behavior because they increased at the end of the dehydration process reaching the maximum contents at P30 for air- and ozone-treated grapes. Higher contents of total free alcohols and aldehydes were observed in grapes dehydrated under ozone-enriched atmosphere from P20 than in fresh grapes. Therefore, the ozone treatment appears to accelerate this increasing trend.

A significant effect of ozone treatment was observed for total free terpenes, alcohols and aldehydes at P30, increasing their contents with respect to untreated samples (air treatment). Terpenes showed significant differences at lower dehydration of grapes (P5) where the ozone treatment (P5-OZ) caused a decrease of total content of free terpenes *versus* air treatment (P5-AR). In the early stages of dehydration (P10), ozone also caused a significant decrease of total content of free alcohols, but it induced an increased content at P20 and P30 *versus* air.

The most abundant free volatile compounds were C_6-_aldehydes, in particular hexanal and (*E*)-2-hexenal, in all stages of dehydration in ozone and air atmosphere. These compounds only showed significant differences among the two treatments at P30. At which time increased contents of free hexanal and (*E*)-2-hexenal were found in ozone-treated samples. Hexanol and (*Z*)-3-hexen-1-ol were the predominant free C_6_-alcohols. Their contents were negatively influenced by the ozone treatment at the beginning of dehydration (P5 for hexanol, P10 for (*Z*)-3-hexen-1-ol), but they increased significantly at the last stages of dehydration (P30 for hexanol, P20 and P30 for (*Z*)-3-hexen-1-ol) as occurred for aldehydes. Other free alcohols, (*E*)-3-hexen-1-ol, benzyl alcohol and octanol, increased their contents at P20 in ozone enriched atmosphere, and also octanol at P30.

Among free terpenes, linalool, geraniol and nerol were the most abundant compounds in Moscato bianco grapes at all levels of dehydration in both ozone and air treatments. Although the berries dehydrated in ozone enriched atmosphere at P5 showed significantly lower contents for most of free terpenes detected (15 from 18), the trend began to change at P20. Ozone induced a higher accumulation of specific compounds (9 from 18) *versus* air at P20 or P30. Moreover, ozone-treated berries showed an increased content of linalool at the end of dehydration (P30) in relation to untreated berries and the same behavior was observed for geraniol and nerol from 20% of weight loss (P20 and P30).

### Glycosylated volatile compounds

Table 3 shows the glycosidically-bound volatile composition of Moscato bianco grapes at different stages of dehydration in ozone enriched atmosphere *versus* air. A total of 32 compounds were quantified, which were grouped in 18 terpenes, 10 alcohols, and 4 aldehydes.

**Table 3:** Glycosidically-bound volatile composition of Moscato bianco grapes treated with ozone (OZ) and air (AR) during postharvest dehydration.

**Bound compounds (µg/L)**	**PO**	**P5-AR**	**P5-OZ**	Sig.	**P10-AR**	**P10-OZ**	Sig.	**P15-AR**	**P15-OZ**	Sig.	**P20-AR**	**P20-OZ**	Sig.	**P30-AR**	**P30-OZ**	Sig.
D-Limonene	21.88	25.86	36.61	ns	24.45	24.85	ns	17.29	21.10	ns	23.98	18.13	ns	12.24	25.73	*
β-Phellandrene	14.02	16.15	16.47	ns	14.13	14.67	ns	13.80	13.11	ns	14.51	13.22	ns	10.63	17.90	**
(*E*)-Ocimene	16.17	19.40	22.13	ns	16.56	17.11	ns	11.78	14.97	ns	16.81	12.86	ns	14.51	19.45	ns
(*Z*)-Ocimene	28.21	33.72	41.55	ns	29.52	30.73	ns	9.47	27.28	*	29.41	22.52	ns	5.64	33.95	**
(+)-4-Carene	6.19	8.27	8.43	ns	6.03	6.24	ns	4.40	5.65	ns	5.82	4.97	ns	6.55	7.92	ns
*trans*-Rose oxide	14.65	26.62	8.92	**	11.28	12.65	ns	9.21	11.01	ns	10.26	13.19	ns	38.31	34.11	ns
*cis*-Rose oxide	4.47	8.29	2.75	**	3.66	3.98	ns	2.95	3.42	ns	3.20	4.21	ns	11.85	10.94	ns
*trans*-Furan linalool oxide	6.68	9.02	5.23	**	7.34	7.50	ns	5.84	8.78	**	8.06	9.99	ns	13.94	15.28	ns
*cis*-Furan linalool oxide	1.02	1.09	0.87	ns	1.10	1.11	ns	0.91	1.04	ns	1.14	1.38	ns	1.79	1.77	ns
Linalool	542.14	588.79	509.21	ns	581.98	547.89	ns	539.08	624.13	ns	580.61	613.37	ns	620.09	754.54	*
Ho-trienol	1.39	1.15	1.97	ns	2.39	1.53	ns	1.99	2.32	ns	2.32	1.49	ns	2.06	1.96	ns
Geranial	56.21	59.66	29.47	***	31.84	28.52	ns	32.18	34.50	ns	37.25	41.46	ns	49.19	46.91	ns
α-terpineol	3.19	3.41	3.20	ns	2.79	2.54	ns	3.01	2.70	ns	3.29	3.40	ns	3.35	3.42	ns
Neral	32.96	36.83	28.35	**	26.58	24.87	ns	28.50	29.55	ns	32.98	35.47	ns	37.02	37.82	ns
*trans*-Pyran linalool oxide	2.37	2.59	2.30	ns	2.60	2.29	ns	2.63	2.67	ns	2.65	3.53	*	4.33	4.61	ns
Citronellol	36.97	48.20	45.79	ns	46.26	36.94	**	45.73	46.86	ns	41.85	48.41	*	55.81	50.65	ns
Nerol	553.07	654.49	669.70	ns	649.03	573.18	ns	665.95	633.16	ns	634.75	720.98	ns	671.23	745.22	ns
Geraniol	450.24	542.06	528.94	ns	498.84	458.14	ns	578.47	532.18	ns	510.13	571.52	ns	500.59	606.16	*
*Bound terpenes*	1791.86	2085.60	1961.90	ns	1956.38	1794.70	ns	1973.21	2014.43	ns	1959.01	2140.08	ns	2059.14	2418.34	**
Isoamyl_alcohol	14.18	15.47	45.39	**	70.68	62.61	ns	74.14	76.26	ns	65.99	46.16	ns	66.27	48.82	ns
Pentanol	3.65	3.17	8.53	***	10.28	10.33	ns	8.09	10.57	*	8.58	7.54	ns	7.65	7.69	ns
3-Methyl-2-buten-1-ol	1.25	1.98	6.73	*	9.36	8.79	ns	3.32	6.88	ns	6.48	3.83	ns	6.16	4.76	ns
Hexanol	81.45	115.12	154.42	**	158.01	172.78	ns	176.91	200.52	ns	161.02	189.35	*	188.61	200.21	ns
*(E)-*3-hexen-1-ol	3.80	6.95	13.95	***	12.56	14.24	ns	12.46	12.11	ns	10.72	10.20	ns	11.51	9.78	ns
*(Z)-*3*-*hexen-1-ol	5.89	11.82	23.51	***	20.83	24.16	ns	23.50	22.42	ns	19.92	20.09	ns	24.06	23.84	ns
2-Ethylhexanol	6.59	7.69	5.77	ns	7.85	7.70	ns	11.26	6.58	**	7.76	10.07	*	10.52	8.04	**
Octanol	9.07	9.58	9.90	ns	8.12	10.33	**	9.58	12.30	**	9.36	14.84	***	10.69	14.73	***
Benzyl alcohol	3.67	3.57	25.46	***	22.70	23.22	ns	12.65	13.62	ns	12.41	8.86	ns	12.87	11.66	ns
2-Phenylethanol	12.76	13.14	34.96	*	32.80	31.28	ns	28.87	28.09	ns	28.73	23.18	***	28.18	27.83	ns
*Bound alcohols*	128.11	173.02	283.23	***	282.52	302.84	ns	286.63	313.10	ns	264.98	287.95	ns	300.24	308.53	ns
Hexanal	5.85	4.27	6.70	*	6.65	7.14	ns	6.92	7.02	ns	8.19	5.89	ns	5.55	9.47	**
Heptanal	1.32	1.46	1.23	ns	1.54	1.49	ns	1.57	2.03	ns	1.49	1.56	ns	1.35	1.75	ns
Octanal	1.56	1.16	1.15	ns	1.61	1.22	ns	1.39	2.16	ns	0.61	2.09	ns	1.55	1.46	ns
Benzaldehyde	1.81	1.79	5.31	**	4.28	4.35	ns	2.22	2.26	ns	3.06	2.29	ns	2.75	2.71	ns
*Bound aldehydes*	10.55	8.69	14.40	*	14.08	14.21	ns	12.10	13.48	ns	13.34	11.83	ns	11.19	15.38	ns

Asterisks denote significant differences between treatments at the same stage of dehydration according to Student’s t-test (P < 0.05, n = 3): *^, ^**and *** indicate significance at *p* < 0.05, *p* < 0.01 and *p* < 0.001, respectively; ns indicates no significant difference. P: sampling point at a defined weight loss (0, 5, 10, 15, 20, and 30%).

In contrast to total free compounds, an increasing trend of total bound terpenes was observed during dehydration for air- and ozone-treated grapes and the maximum contents were found at P30. Total bound alcohols and aldehydes also increased during dehydration.

Significant differences were found in total content of glycosylated terpenes in grapes dehydrated at P30 in ozone *versus* air. However, the differences in total content of glycosylated alcohols and aldehydes were observed at P5. In either case, ozone promoted the increase of glycosylated volatile compounds.

The predominant glycosylated volatile compounds were terpenes, in particular linalool, geraniol and nerol, at all levels of dehydration in both ozone and air treatments. Berries dehydrated at P5 in ozone enriched atmosphere showed a significant decrease of the contents of a few non-major glycosylated terpenes quantified (5 from 18) in relation to air. Glycosylated linalool and geraniol were significantly affected by the ozone treatment at P30, increasing their contents *versus* control samples. Ozone influenced positively nerol by increasing its content at P20 and P30, but the differences were not significant when compared with untreated berries. The ozone presence also induced a significant increase of the content of other bound terpenes ((*Z*)-ocimene, β-phellandrene, and D-limonene) at P30. It is important to take into account that the ozone treatment makes it possible to exceed the content found in fresh grapes (PO) for all glycosylated terpene detected, with the exception of geranial.

Among glycosylated alcohols, hexanol was the most abundant volatile compound. Most of alcohols (8 from 10, including hexanol) were found in significantly higher contents at P5 in ozone enriched atmosphere. Octanol showed an increased content in ozone-treated berries through the dehydration process.

Regarding aldehydes, hexanal was the major glycosylated compound and exhibited a significant increase of its content at the early (P5) and the last (P30) stages of dehydration in ozone enriched atmosphere *versus* air. Similar behavior was showed by benzaldehyde only at the beginning of the postharvest dehydration process (P5).

### Differentiation of postharvest ozone-treated grapes according to terpene composition

**Figure 2:**
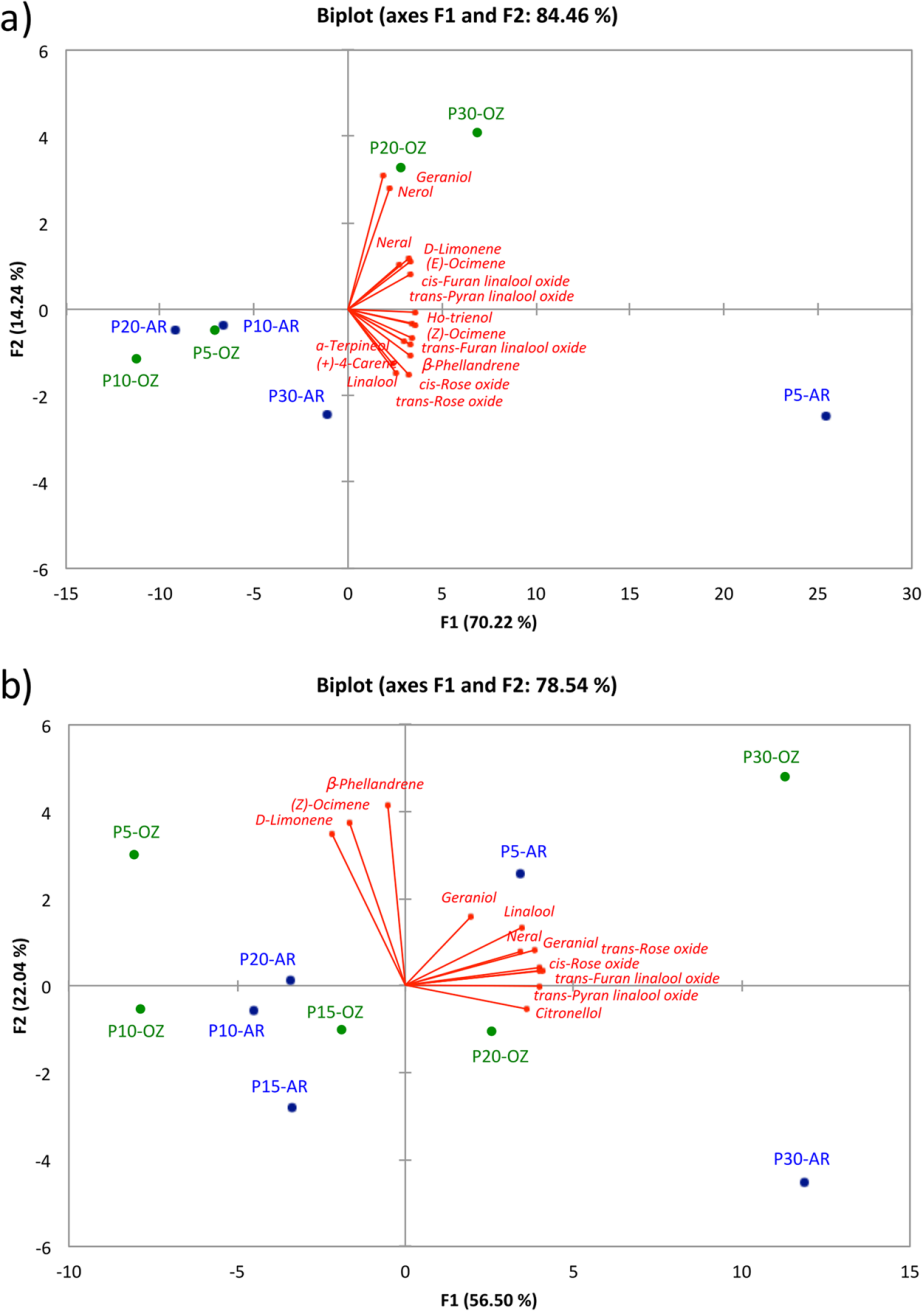
Principal components analysis (PCA) applied to the terpene composition of dehydrated Moscato bianco grapes. The PCAs exhibit the distribution of samples dehydrated in ozone enriched atmosphere (OZ) and air (AR) according to free (**a**) and glycosylated (**b**) terpene composition. P0, P5, P10, P15, P20, and P30 indicate sampling point at a defined weight loss of 0, 5, 10, 15, 20, and 30%, respectively.

Given the importance of terpenes in the Moscato bianco cultivar, Principal Components Analyses (PCAs) were performed on terpene composition to better visualize the differentiation of grape berries dehydrated in ozone enriched atmosphere from those dehydrated in air (Fig. 2). PCAs were conducted on free and glycosylated terpenes with significantly different contents among samples.

Figure 2a corresponds to the PCA conducted on free terpenes. The first two principal components (PC1 and PC2) accounted for 84.46% of the total variance (70.22% and 14.24%, respectively). The P15-OZ and P15-AR samples were not represented in the plot because no significant differences were found in free terpene composition. Three well-defined groups can be differentiated. The first group is formed by the more dehydrated samples in ozone enriched atmosphere (P20-OZ and P30-OZ), which are sited in the positive side of both PC1 and PC2. The second group includes only the less dehydrated berries in air (P5-AR, positive side of PC1 and negative side of PC2), and the third group encompasses the more dehydrated samples in air and those less dehydrated in ozone (negative side of PC1 and PC2). All terpenes were highly correlated with PC1, with the exception of nerol and geraniol that contributed mainly to PC2.

Figure 2b shows the PCA conducted on glycosylated terpenes. The first two principal components (PC1 and PC2) accounted for 78.54% of the total variance (56.50% and 22.04%, respectively). The samples were clustered into three groups corresponding to different treatments. More dehydrated grape samples in ozone enriched atmosphere (P30-OZ) are associated with the highest positive values of PC1 and PC2. They are well differentiated from the corresponding control (P30-AR), which is sited in the highest positive values of PC1 but the highest negative values of PC2. The third group, formed by the other samples, is located in the intermediate and highest negative values of PC1. The PC1 was associated with the contents of *trans*-rose oxide, *cis*-rose oxide, *trans*-furan linalool oxide, linalool, neral, *trans*-piran linalool oxide, citronellol and geranial whereas the PC2 was strongly correlated with D-limonene, β-phellandrene and (*Z)*-ocimene.

Therefore, the grape berries dehydrated at P30 in ozone enriched atmosphere (P30-OZ) were well differentiated and characterized by high contents of free and glycosylated terpenes, with the exception of glycosylated geraniol with low representation in the two components.

### Monoterpene biosynthetic pathway

**Figure 3:**
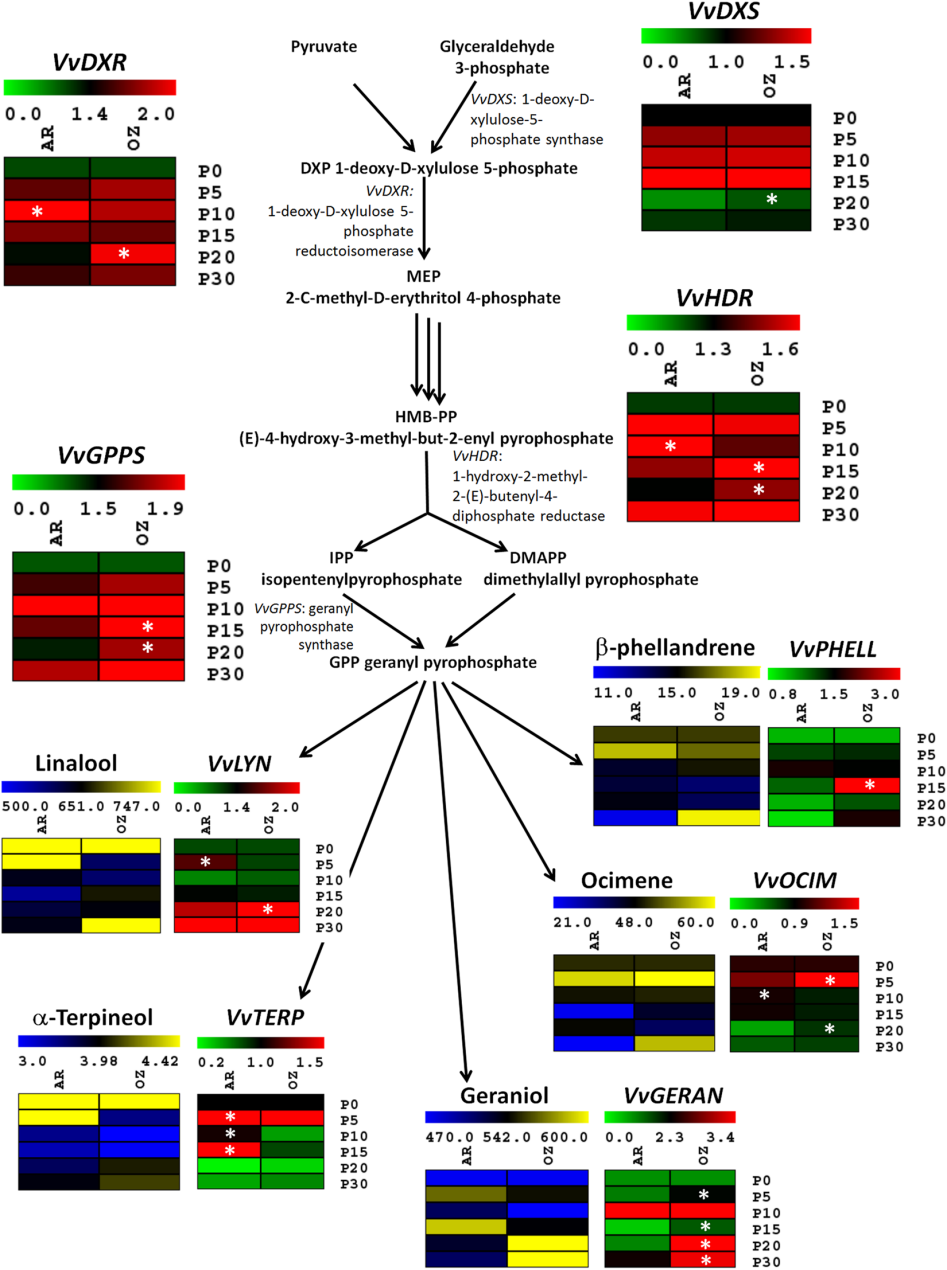
Transcriptional modulation of genes involved in methylerythritol phosphate (MEP) pathway. Relative expression levels obtained by RT-qPCR analysis of the *VvDXS*: 1-deoxy-D-xylulose-5-phosphate synthase (VIT_05s0020g02130); *VvDXR*: 1-deoxy-D-xylulose 5-phosphate reductoisomerase (VIT_17s0000g08390); *VvHDR*: 1-hydroxy-2-methyl- 2-(E)-butenyl-4-diphosphate reductase (VIT_03s0063g02030); *VvGPPS*: geranyl pyrophosphate synthase (VIT_15s0024g00850); *VvLYN*: linalool synthase (VIT_00s0271g00060); *VvGERAN*: geraniol synthase (VIT_12s0134g00140); *VvTERP*: terpineol synthase (VIT_13s0084g00010); *VvOCIM*: ocimene synthase (VIT_12s0134g00030); *VvPHELL*: phellandrene synthase (VIT_13s0067g03830). Genes were tested during the different dehydration stages under air (AR) and ozone (OZ) treatments. P0, P5, P10, P15, P20, and P30 indicate sampling point at a defined weight loss of 0, 5, 10, 15, 20, and 30%, respectively. The results were represented by heatmap green-red corresponding respectively to low and higher transcriptional levels. The levels of single monoterpene (free and glycosylated) were represented by heatmap yellow-blue representing respectively low and higher accumulation of the metabolite. Asterisks denote significant differences between treatments at the same stage of dehydration as attested by Student’s *t*-test (P < 0.05).

Since the ozone treatment induced a significant increase of total monoterpenes content in the last stages of postharvest dehydration (Fig. 1), the transcription levels of some key genes involved in MEP pathway, critical for monoterpenes biosynthesis in grape berries, were analyzed. The first gene of the pathway, 1-deoxy-D-xylulose-5-phosphate synthase (*VvDXS*)^35^, showed a progressive increase of transcription up to P15 in both the conditions (air and ozone), followed by a strong reduction at P20 and P30 (Fig. 3). However, the levels of *VvDXS* remained significantly higher at P20 in the presence of ozone (Fig. 3). Similarly, the following genes of MEP pathway, 1-deoxy-D-xylulose-5-phosphate reductoisomerase (*VvDXR*) and 1-hydroxy-2-methyl-2-(*E*)-butenyl-4-diphosphate reductase (*VvHDR*)^7^, showed an increase of transcription in the early phases of dehydration, but with a different trend in the two conditions (Fig. 3). In air, for both *VvDXR* and *VvHDR* genes we observed higher transcription at P10, followed by a decrease in the last stages of dehydration. However, in the presence of ozone, the transcription levels increased at P5 and remained constant in the following dehydration stages. In this way, the expression of *VvDXR* and *VvHDR* was significantly higher at P20-OZ in comparison to P20-AR (Fig. 3). The geranyl pyrophosphate synthase (*VvGPPS*), a MEP mid-pathway gene that produces geranyl pyrophosphate as substrate of monoterpenes synthases in the late pathway, showed general increased transcription when dehydration started, and in particular at P15 and P20 the ozone induced a significantly higher activation of this gene (Fig. 3).

The expression changes of these four genes during dehydration support the accumulation of total terpenes reported above (Fig. 1). In particular, the precocious transcription activation in the early phases of dehydration in air suggests the increase of terpene contents at P5-AR and P10-AR, although not always significant (Figs 1 and 3, Tables 1 and 2). Similarly, the general up-regulation of these genes at P15-OZ and P20-OZ likely determined the increment of terpenes in the presence of ozone at the end of the dehydration process (Figs 1 and 3, Tables 1 and 2).

In addition, five genes involved in the late MEP pathway in the production of specific monoterpenes were analyzed, allowing a correlation between transcripts and metabolites. A linalool/nerolidol synthase (*VvLYN*)^7^ showed a significant transcription increase at P5-AR and a higher activation at the end of postharvest dehydration, in particular at P20-OZ. The total content of free and glycosylated linalool reflects the transcription levels of *VvLYN* (Fig. 3). The total contents of geraniol (free and glycosylated forms) and the transcription of a geraniol synthase (*VvGERAN*)^36^ agreed at the last stages of the dehydration process; higher contents at P20-OZ and P30-OZ were associated with higher transcriptional levels of *VvGERAN* between P15-OZ and P30-OZ (Fig. 3). Unlike other terpenes, the total contents of α-terpineol generally decreased during dehydration in both ozone and air atmosphere, reaching higher levels at P5-AR. The α-terpineol synthase (*VvTERP*)^7^ showed a transcription increase in the early phases of dehydration (P5-P15) in particular in air conditions, followed by a progressive decline at higher levels of water loss (Fig. 3). The phellandrene synthase (*VvPHELL*)^36^ had a high peak of expression at P15-OZ and a higher accumulation of the metabolite at P30-OZ (Fig. 3). For the ocimene synthase (*VvOCIM*)^36^, we observed a low correspondence with the metabolite: after a precocious induction in particular for ozone-treated berries at P5-OZ, the transcription decreased as dehydration progressed whereas the ocimene isomers showed an increased accumulation not only at P5-AR and P5-OZ but also at P30-OZ (Fig. 3).

### Lipoxygenase–hydroperoxide lyase (LOX–HPL) pathway

In addition to monoterpenes, the ozone treatment induced an increased total content of C_6_ compounds, in particular aldehydes at the last stages of dehydration (Fig. 1). The transcription levels of some important genes involved in the ROS metabolism and the LOX–HPL pathway were analyzed. Taking into account that the over-production of ROS in response to ozone has been reported in green tissues^23^, and that the ROS accumulation, among other reactions, acts as a signal for PUFAs formation^37^, we verified if the ozone also affects the ROS metabolism in dehydrated grape berries.

**Figure 4:**
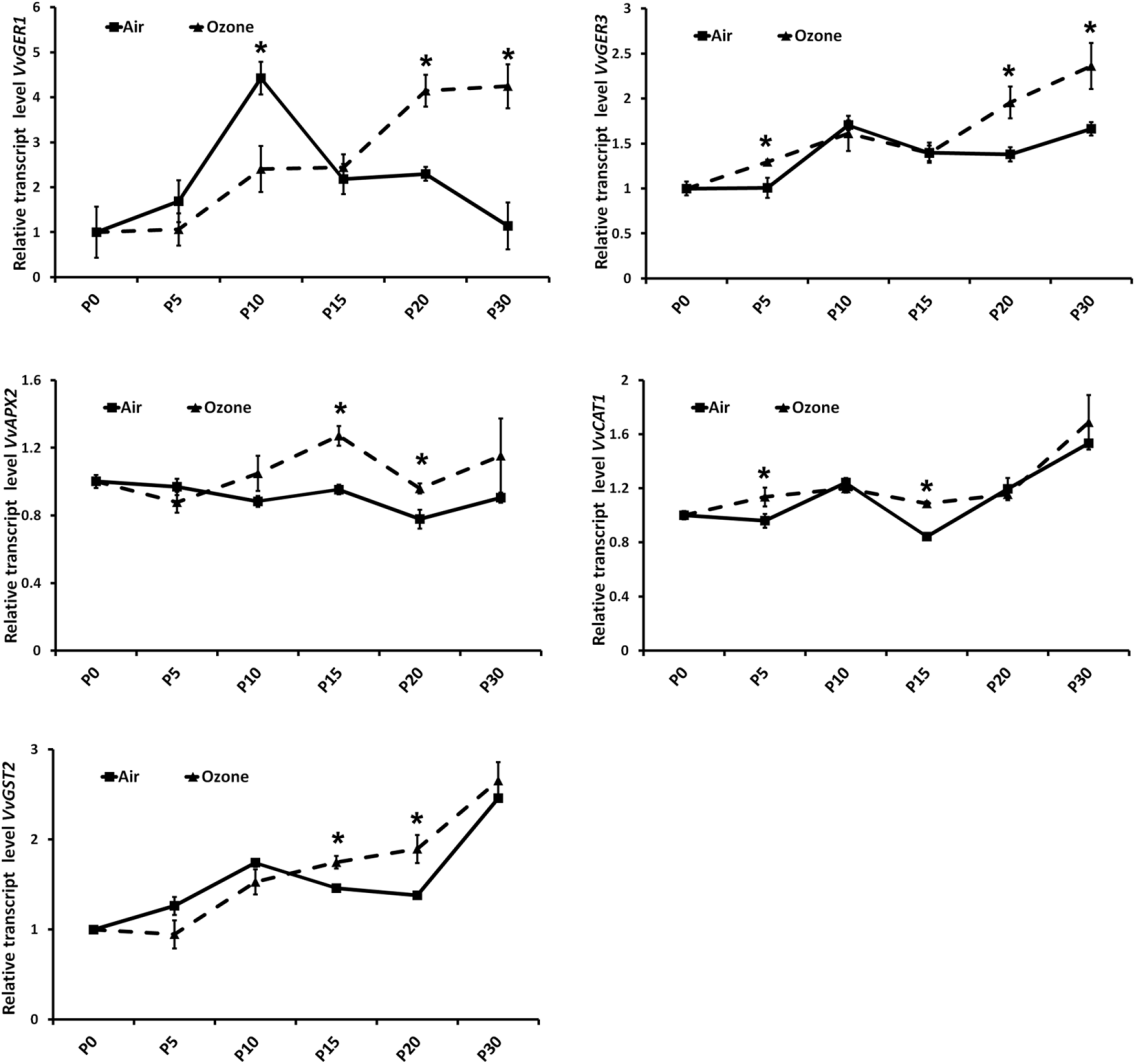
Transcriptional modulation of genes involved in reactive oxygen species (ROS) metabolism. Relative expression levels obtained by RT-qPCR analysis of the *VvGER1* (**a**): germin-like protein 1 (VIT_14s0128g00670); *VvGER3* (**b**): germin-like protein 3 (VIT_14s0128g00690); *VvAPX2* (**c**): ascorbate peroxidase (VIT_08s0040g03150); *VvCAT1* (**d**)*:* catalase (VIT_18s0122g01320); *VvGST2* (**e**): glutathione S-transferase (VIT_16s0013g01500). Genes were tested during the different dehydration stages under air (AR) and ozone (OZ) treatments. P0, P5, P10, P15, P20, and P30 indicate sampling point at a defined weight loss of 0, 5, 10, 15, 20, and 30%, respectively. Asterisks denote significant differences between treatments at the same stage of dehydration as attested by Student’s *t*-test (P < 0.05).

Two germin-like transcripts (*VvGER1* and *VvGER3*) involved in the production of H_2_O_2_ and activated in response to pathogens were strongly overexpressed at P20 and P30 in the presence of ozone when compared with air (Fig. 4). In addition, scavenging genes, such as catalase (*VvCAT1*) and ascorbate peroxidase (*VvAPX2*), involved in the detoxification of H_2_O_2_, and glutathione S-transferase (*VvGST2*) involved in the oxidative stress prevention were significantly up-regulated in the presence of ozone, in particular *VvAPX2* and *VvGST2* at P15 and P20 (Fig. 4). These data confirmed the previous observation in photosynthetic tissues^23^ and suggest an over-accumulation of ROS during the dehydration process, in particular in the presence of ozone.

**Figure 5:**
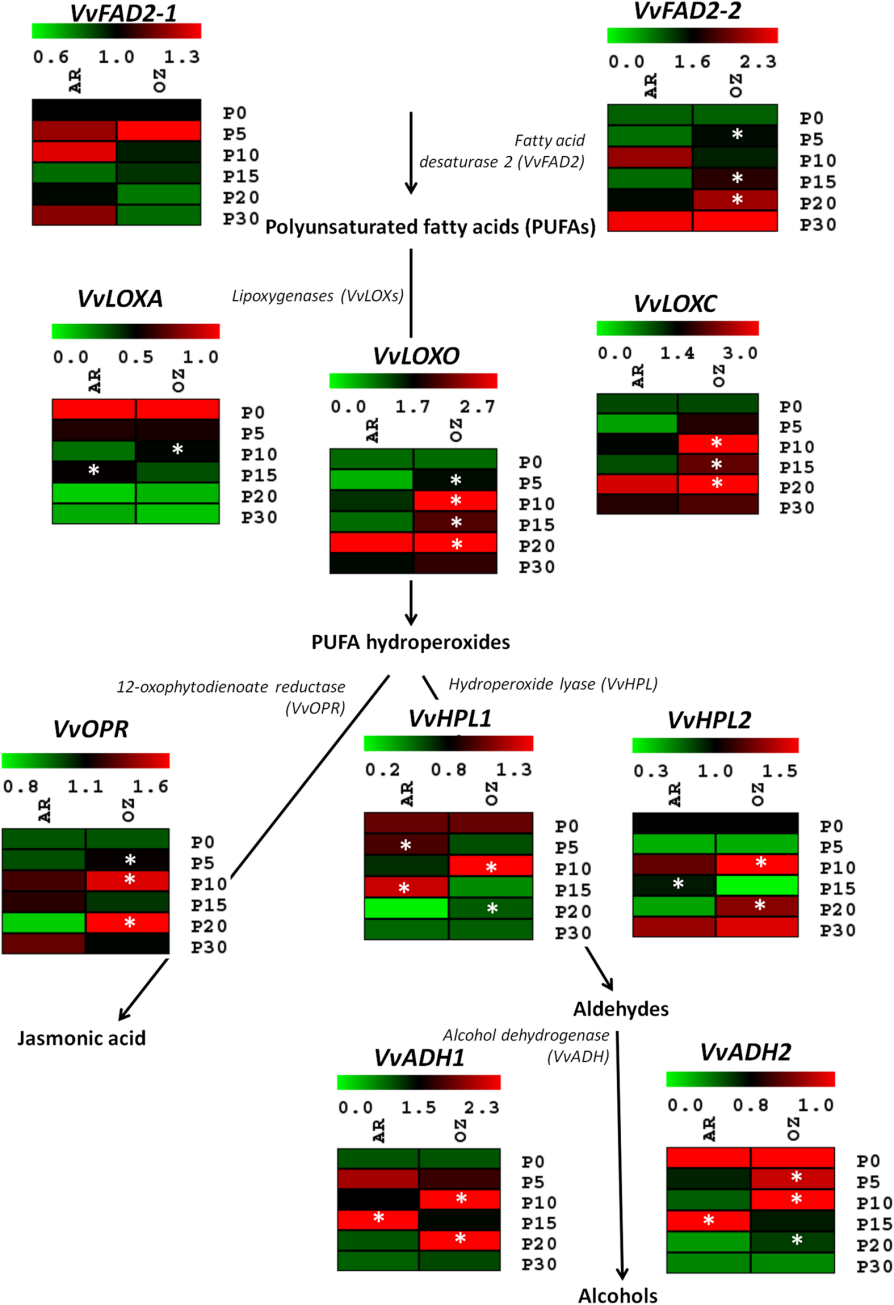
Transcriptional modulation of genes involved in lipoxygenase–hydroperoxide lyase (LOX–HPL) pathway. Relative expression levels obtained by RT-qPCR analysis of the *VvFAD2-1*: fatty acid desaturase 2 (VIT_08s0007g06450); *VvFAD2-2*: fatty acid desaturase 2 (VIT_06s0004g01250); *VvLOXA*: lipoxygenase (VIT_06s0004g01510); *VvLOXO*: lipoxygenase (VIT_09s0002g01080) *VvLOXC*: lipoxygenase (VIT_14s0128g00780; VIT_14s0128g00790); *VvHPL1*: hydroperoxide lyase (VIT_12s0059g01060); *VvHPL2*: hydroperoxide lyase (VIT_03s0063g01830); *VvADH1*: alcohol dehydrogenase (VIT_18s0001g15410); *VvADH2*: alcohol dehydrogenase (VIT_04s0044g01110); *VvOPR*: 12-oxophytodienoate reductase (VIT_11s0016g01230). Genes were tested during the different dehydration stages under air (AR) and ozone (OZ) treatments. P0, P5, P10, P15, P20, and P30 indicate sampling point at a defined weight loss of 0, 5, 10, 15, 20, and 30%, respectively. The results were represented by heatmap green-red corresponding respectively to low and higher transcriptional levels. Asterisks denote significant differences between treatments at the same stage of dehydration as attested by Student’s *t*-test (P < 0.05).

Two fatty acid desaturase 2 genes (*VvFAD2-1* and *VvFAD2-2*) involved in PUFAs synthesis^38^ exhibited a different expression pattern in the two experimental conditions (air and ozone). The *VvFAD2-1* did not show significant differences of expression between ozone- and air-treated berries. Although the expression of *VvFAD2-2* increased during dehydration in both the conditions (Fig. 5), the ozone induced an overexpression of this gene at P5, P15 and P20 in agreement with the increase of total aldehyde contents at the end of dehydration (Figs 1 and 5).

In the following step of the pathway, the PUFAs are oxygenated by LOXs producing 13-carbon and 9-carbon hydroperoxide precursors of several oxylipins (C_6_ and C_9_ compounds, and also jasmonic acid). Two 13-*LOXs* (*VvLOXA* and *VvLOXO*) and one 9-*LOX* (*VvLOXC*) genes, previously identified in grapevine^11^, were analyzed. The *VvLOXA* showed a general down regulation during dehydration and does not seem to be influenced by stresses (dehydration and ozone) or to be strongly correlated with the production of C_6_ compounds in grape berries (Fig. 5). Conversely, both *VvLOXO* and *VvLOXC* were strongly activated during the dehydration process and in particular in the presence of ozone at P10, P15, and P20 (Fig. 5).

In the following step of aldehydes production catalyzed by hydroperoxide lyase (HPL), we observed a high expression of *VvHPL1* at P10-OZ and in particular of *VvHPL2* at P10-OZ and P20-OZ (Fig. 5), which agreed with the higher accumulation of aldehydes (Fig. 1). The *VvADH1* and *VvADH2*, involved in the conversion of aldehydes to alcohols, showed a transcription modulation quite similar to *VvHPL*. In particular, a peak of expression was observed for *VvADH2* at P5-OZ and P10-OZ (Fig. 5). The PUFA hydroperoxides are also precursors of jasmonic acid (JA). We analyzed the expression of 12-oxophytodienoate reductase (*VvOPR*), which is one of the most important genes involved in JA biosynthesis. The ozone treatment during the dehydration process induced a strong expression of this gene in particular at P10-OZ and P20-OZ (Fig. 5). This suggests that the berries even in post-harvest conditions react to stresses likely activating JA-mediated defense responses.

## Discussion

Heath^23^ reported that multiple metabolic pathways can be stimulated by ozone, depending on dose and exposure time. Other previously published works showed that ozone treatment does not affect the content of reducing sugars and titratable acidity in Petit Verdot grapes after cold treatment overnight^30^. In agreement with our results, short-term ozone-exposed samples showed a significantly higher content of total soluble solids and lower value of titratable acidity than those unexposed to ozone enriched atmosphere for Grechetto grapes^39^. In tomato fruit and papaya, increased contents of soluble solids and glucose were also found in long-term ozone-treated fruits^40,41^.

Regarding organic acids, the lower initial content of malic acid in ozone-treated berries probably due to increased respiration and even gluconeogenesis could have been overturned at P15. This agreed with the double stress response proposed by Botondi *et al*.^32^, who suggested that the malate catabolism is affected by water loss and ozone treatment especially in the case of long-term treatments of grapes. A similar behavior was also observed for tartaric acid at P10 because of its lower susceptibility to catabolism. Nevertheless, the lower contents of citric acid in ozone-treated samples, when they were dehydrated at P15 and P30, agreed with the results found in kiwi fruit during cold storage in ozone enriched air^42^.

Organic acids play an important role in wine aroma compounds. The release of stored organic acids, specifically L-malic acid and tartaric acid, from the grape berry during crushing is responsible for acid hydrolysis of glycosylated compounds such as monoterpenes, C_13_-norisoprenoids, benzyl alcohol, and 2- phenylethanol^43^.

Biotic and abiotic stressors affect volatile compounds production in plants^44^. In grapevine, abiotic stress modifies the growth and development of all plant organs. At the berry level, abiotic stress induces the accumulation of secondary metabolites in the pulp, seeds and skins as a defensive response against cell damages. Viticulture practices can be managed to control the plant response to stress with the purpose of increasing the content of secondary metabolites and therefore enhancing grape and wine quality, in particular taste and aroma^45^.

The results presented here show how the ozone treatment can affect the synthesis of VOCs, mainly monoterpenes, alcohols and aldehydes, during postharvest dehydration, improving the Moscato bianco grape quality. Terpenes are the volatile compounds responsible for the characteristic varietal aroma of grapes, in particular in white cultivars belonging to Muscat family^46,47^. Moscato bianco is an aromatic cultivar characterized by a terpene profile composed mainly of linalool, geraniol, and nerol^48^. These compounds, present in free and glycosidically-bound forms, have low perception thresholds and therefore contribute strongly to the grape aroma quality with fruity and floral nuances^49,50^.

Terpene metabolites are implicated in several ecological and physiological functions on the basis of the differential expression profiles of terpene synthase genes observed throughout plant development and in response to biotic and abiotic environmental factors^51^. Abiotic stressors, such as temperature changes, UV-B radiation, water deficit, basal leaf removal or crop thinning, influence grape and wine terpene composition^52,53^. Monoterpenes and C_6_-compounds are produced in plants also as response to ozone-stress treatment^19^.

During dehydration, De Sanctis *et al*.^31^ observed that glycosylated volatile compounds increased in ozone-treated Sauvignon blanc grapes *versus* untreated ones whereas the free volatiles were higher in untreated grapes. A similar behavior was observed in the present study for total glycosylated volatile compounds. Our results showed an increase in total content of glycosylated terpenes from 15% of water loss, whereas increased total contents of glycosylated alcohols and aldehydes were evident already from 5% of water loss, although the differences were not always significant between both ozone and air treatments (Table 3). However, we observed a significant decrease of total contents of free terpenes and alcohols only in the first stages of dehydration in ozone *versus* air (P5 for terpenes and P10 for alcohols), this trend being reversed at the end of dehydration (Table 2). In fact, ozone-treated grapes were significantly richer in total free terpenes at P30 and in total free alcohols at P20 and P30 than control samples (untreated, air-treated). As a consequence, total VOC contents were significantly higher in the last stages of dehydration (P20 and P30) in ozone enriched atmosphere *versus* control samples (Fig. 1). Therefore, the long-term ozone treatment promoted these increased contents of free VOCs in relation to shorter exposure times to ozone. De Sanctis *et al*.^31^ used ozone treatments lasting only 16 h.

It is important to highlight that total content of glycosylated terpenes was higher than total free fraction at all levels of dehydration. Battilana *et al*.^54^ reported that the free and bound forms of monoterpenes have different accumulation profiles during berry ripening. Moscato bianco showed the maximum content of free fraction at harvest, but a continued increase of glycosylated fraction was observed in overripe grapes. Torchio *et al*.^48^ also confirmed these differences in the evolution of total content of free and bound terpenes during ripening of Moscato bianco grapes.

In grape berries, monoterpenes are predominantly in glycosylated forms^49^. Possible functions of glycosylation are sequestration, detoxification, and decrease of volatility and reactivity because the free volatiles may be toxic at high concentrations to the plant itself^55^. Therefore, in our work, ozone abiotic stress could induce the increase of free volatiles during dehydration and then they could be accumulated in berries mainly as glycosylated derivatives. Glycosides are not volatile and therefore they do not directly contribute to wine aroma. However, they affect indirectly the aroma because aglycones can be released during fermentation by yeasts and bacteria or even by the addition of exogenous glycosidases, increasing the final wine aroma^56^.

The increase of terpene glycosylation at P30 in ozone enriched atmosphere *versus* air agrees with the decrease of reducing sugars content (glucose and fructose). In the glycosylation process, the first sugar directly attached to the aglycone is in all cases glucose, which could explain this loss of glucose. In the case of terpenoids, the majority of glycosides are present in diglycoside form, in which a second sugar (arabinose, apiose, or rhamnose) is attached to the glucose^56^.

On the other hand, the presence of *Botrytis cinerea* in grapes can cause the degradation of the main monoterpene alcohols and their oxides into generally less odorous components^57,58^. Therefore, the use of ozone as sanitizing agent on grapes to control fungi development during dehydration could help to preserve the aroma quality of the grapes. Nevertheless, Blanco-Ulate *et al*.^59^ showed that the biosynthesis of terpenes and fatty acid aroma precursors increases during noble rot by *Botrytis cinerea* infection. Noble rot causes a major reprogramming of berry development and metabolism, leading to a greater accumulation of compounds involved in the unique flavor and aroma of botrytized wines. Nevertheless, these authors highlighted that the accumulation of terpenes might not be a characteristic of the *Botrytis cinerea* infection but only of noble rot. At the beginning of dehydration (P5), we also observed significantly higher contents of total free terpenes and some bound terpenes (*trans*-rose oxide, *cis*-rose oxide, *trans*-furan linalool oxide, geranial and neral) when grapes were air-treated, and therefore more exposed to *Botrytis cinerea*. However, the increase of these compounds was reported in ozone-treated grapes at the end of the dehydration process.

The *de novo* biosynthesis of monoterpenes in grapes via MEP pathway has been demonstrated^6^. Analyzing some of the major genes involved in this pathway, which were previously identified in grapevine^7,36^, we showed that likely the increase of monoterpenes production at P30-OZ was linked to the over-expression of these genes. The grapevine berries at postharvest, although detached from the plant, continue to be metabolically active with the modulation of several transcripts^33^, and to respond actively to environmental conditions. Indeed, in this study, we observed a general up-regulation of MEP pathway in partially dehydrated grapes, in particular the increment associated with the application of a second stressor, the ozone (Fig. 3).

*VvHDR* and *VvGPPS* are the genes that showed the highest correlation with the metabolic data, confirming the previous observation in Gewürztraminer grapes^7^ and in *Arabidopsis* ^60^. In our work, *VvHDR* in Moscato grapes seems to have an important role in controlling the production of MEP-derived precursors. The transcriptional regulation of this gene reflected the higher accumulation of monoterpenes in air-treated grape berries at P5 and P10 (even if not significant in some cases), but an increase of these metabolites was observed at P20 and P30 in the presence of ozone (Figs 1 and 3). Although *VvDXS* is a pivotal gene for the production of monoterpenes in grapes, and in particular for the Muscat flavour formation^54^, in our experiment its transcriptional regulation did not agree with the increase of monoterpene contents during dehydration in ozone-enriched atmosphere. Similar observations were reported previously in Gewürztraminer grapes^7^. However, *VvDXS* showed a rapid activation in the first phases of dehydration (from P5 to P15), suggesting a precocious response to abiotic stresses, followed by the increased expression of the downstream genes. Indeed, those other genes (*VvDXR*, *VvHDR*, and *VvGPPS*) had a peak of expression slightly later in the presence of ozone (P15 and P20). The two terpene synthases (*VvLYN* and *VvGERAN*)^7,36^, which are responsible for the production of linalool and geraniol, showed the maximum expression at the end of dehydration (P20 and P30) in ozone enriched atmosphere (Fig. 3).

In grapes, the major volatile compounds derived from fatty acids are C_6_-aldehydes and alcohols^61,62^, many of which are responsible for “fresh green” aromas in grape juice. C_6_-compounds are synthesized by oxidative cleavage of PUFAs. (*Z*)-3-Hexenal and (*E*)-2-hexenal are derived from linolenic acid pathway (Route A) while hexanal is produced from linoleic acid (Route B). Finally, ADH reduces the aldehydes to the corresponding alcohols, namely hexanol, (*Z*)-3-hexenol and (*E*)-2-hexenol^63,64^. Nevertheless, (*Z*)-3-hexenol may be formed from (*E*)-3-hexenal, which derives from (*Z*)-3-hexenal as referred by Hatanaka^64^ for various fruits.

The determination of free C_6_-compounds in dehydrated grapes showed that free hexanal was richer than free *(E)-*2-hexenal, which indicated that linoleic acid pathway (Route B) predominated over linolenic acid pathway (Route A) during berry dehydration. In addition, both routes (A and B) were more active when the grapes were treated with ozone in all levels of dehydration, although the differences were only significant at the greatest water loss (P30) when compared with control samples. Similar behavior was observed in glycosylated C_6_-compounds, in particular hexanal (Route B) exhibited higher contents than (*E*)-2-hexenal (Route A).

For bound C_6_-compounds, ozone induced a significant increase of their contents *versus* air at the first stage of dehydration (P5, Table 3). This situation could be attributed to membrane denaturation as the first signal of ozone damage. Similar results were observed in ozone-stressed leaves, in which C_6_-compounds were emitted as response to the ozone absorbed by the leaves^37^. This signal is likely linked to the accumulation of ROS induced by ozone, as previously reported in leaves of several plants^20,65^. Interestingly, we observed a transcriptional activation of several genes involved in ROS metabolism (*VvGER*, *VvAPX2*, *VvCAT1*, and *VvGST2*) in postharvest (off-vine) dehydrated grapevine berries (Fig. 4), similar to the response observed in photosynthetic tissues. The ROS accumulation acts as a signal for PUFAs formation^37^, activating some genes involved in the synthesis of PUFAs. For example, in our conditions, the gene *VvFAD2-2* ^38^ showed a progressive increase of transcription during grape dehydration, in particular in the presence of ozone, suggesting a transcriptional regulation modulated by abiotic stresses (Fig. 5). Conversely, another fatty acid desaturase (*VvFAD2-1*) analyzed^38^ showed an expression almost constitutive in our conditions (Fig. 5). Similar regulation was observed for *VvLOXA*, which is a lipoxygenase important for berry ripening^66^. However, in our study on dehydrated Moscato bianco berries, *VvLOXA* modulation does not appear sensitive to stresses. Instead, the transcription of *VvLOXO* was highly activated in the presence of ozone (Fig. 5), suggesting an interesting correspondence with the accumulation of C_6_-compounds (Fig. 1). It confirmed the previous observation on the up-regulation of this gene in the presence of stresses, as for example the infection by *B. cinerea* ^11^. Similar transcriptional regulation and sensitivity to ozone application was observed also for *VvLOXC* (Fig. 5), which is linked to the production of C_9_-compounds. Other genes involved in the production of aldehydes and alcohols, such as *VvHPLs* ^12^ and *VvADHs* ^9^, showed variable expression levels with peaks for both ozone and air conditions in the different dehydration phases (Fig. 5). Accordingly, the three genes, whose expression is better correlated with a general over-accumulation of aldehydes during dehydration, were *VvFAD2-2, VvLOXO*, and *VvLOXC*. Therefore, they could be used as transcriptomic markers for the precocious prediction of the over-production of these compounds in grape berries, in particular under abiotic stress conditions (water loss and ozone).

Since the LOX activity produced precursors not only for C_6_-compounds, but also for the synthesis of JA, we observed an overexpression in ozone-dedydrated grapes of *VvOPR* transcription (Fig. 5), one of the most important genes involved in JA biosynthesis. This suggests that the microbial sanitization in fruits and vegetables, and the reduction of spoilage microflora in grapes^28,40^ after ozone treatments could be partially linked to the activation of JA-mediated defense responses.

In both MEP and LOX-HPL pathways, we observed a delay among the levels of gene transcription and metabolites accumulation. Indeed, many genes showed a peak of transcription at P15 and P20, followed by a decrease at P30, while the peak of accumulation for the corresponding metabolite was mostly observed at P30. This can be explained because the transcription of genes temporarily precedes the production of metabolites. Therefore, it is reasonable to suppose that the changes of transcription at P20 were detectable at metabolic level at P30. Likewise, the overall transcription reduction at P30 may be related to greater dehydration and to age of berry, which could adversely affect the metabolic response of the berry.

Our findings provide important information on the impact of ozone on the synthesis of terpenes and C_6_ compounds during postharvest grape dehydration. Besides the traditional use of ozone as sanitizing agent on postharvest grape berries, our results suggest that controlled applications of this oxidative stressor during long-term treatments can lead to relevant transcriptional changes even in detached berries. As a consequence of these changes, an increased synthesis of many volatile compounds occurs, which are of great importance for grape quality and wine aroma. Specific transcriptomic markers can be used for the over-production prediction of terpenes and C_6_ compounds.

## Methods

### Grapes and dehydration process

Whole bunches of *Vitis vinifera* L. cv. Moscato bianco grapes were harvested in 2015 at a vineyard located in the Piedmont wine region (Asti province, North-West Italy) when 27.0 ± 0.2°Brix were reached in the different sampling zones. About 1 kg of grape berries were randomly selected for fresh grape analysis (P0). Healthy whole bunches were divided in smaller clusters and then arranged in a single layer into ten perforated boxes (30 cm × 20 cm, about 2 kg of grape berries per box) for correct aeration. The grape withering process was performed at 20 ± 2 °C and 60 ± 5% relative humidity (RH)^67^ into thermohygrometrically controlled chambers for 24 days under air (AR, control samples, five boxes) and ozone-enriched (30 µL/L)^68^ atmosphere (OZ, treated samples, the other five boxes). The environmental conditions were constantly monitored and recorded in the two withering chambers using a data logger (HOBO H8 RH/Temp, Onset Computer Corporation, Bourne, MA). For the sample treatment, ozone was continuously supplied by an ozone generator (C32-AG, Industrie De Nora Spa, Milan, Italy) with a nominal production capacity of 32 g O_3_/h. A BMT 964 UV-photometric ozone analyzer (BMT Messtechnik Gmbh, DE) controlled the ozone generator output by continuously monitoring the ozone concentration in the chamber through the recirculation of the ozone-enriched air (120 m^3^/h flow). About 1 kg of AR and OZ grape samples were randomly taken at 5, 10, 15, 20 and 30% of weight loss (P5, P10, P15, P20 and P30, respectively) for withered grape analysis.

### Chemical analysis

For each sample (fresh and differently withered grapes), three replicates of about 100 berries were randomly selected. In the grape juice obtained by manual crushing and centrifugation from each replicate, pH was determined by potentiometry using an InoLab 730 pH meter (WTW, Weilheim, DE). Titratable acidity (g/L tartaric acid) was determined using the International Organization of Vine and Wine method^69^. A high-performance liquid chromatography (HPLC) system (Agilent Technologies, Santa Clara, CA, USA) was used to determine reducing sugars (glucose and fructose) (g/L) with a refractive index detector and tartaric acid, malic acid, citric acid and glycerol (g/L) with a diode array detector (DAD) set to 210 nm^70^.

### Extraction and determination of volatile compounds

Three replicates of 100 g of grape berries were randomly selected for the determination of free and glycosylated volatile compounds in each sample of fresh and differently withered grapes. The grape berries were treated following the method reported by Rolle *et al*.^71^. For each replicate, the grape juice was obtained by crushing the berries for 1 min under a nitrogen atmosphere with a laboratory blender (Waring Laboratory, Torrington, CT, USA) and centrifugation (7000 x g, 15 min, 4 °C). A 5-mL aliquot was diluted with 5 mL of deionized water, adjusted to pH 5 and placed into a 20-mL glass headspace sampling vial containing 2 g of sodium chloride. A 1.55 mg/L of 1-heptanol solution in 10% v/v ethanol was then added as internal standard (200 µL).

The extraction of glycosylated volatile compounds was carried out according to the method proposed by Wang *et al*.^72^ with some modifications. Briefly, 10 mL of the grape juice were loaded onto a 1 g Sep-Pak C18 cartridge (Waters Corporation, Milford, MA, USA). The free fraction was eluted with 10 mL of dichloromethane, and the cartridge was then washed with 10 mL of deionized water. The glycosylated fraction was recovered with 10 mL of methanol, evaporated to dryness using a vacuum rotavapor (Buchi R-210, Switzerland) at 35 °C and dissolved in 5 mL of 0.2 M citrate-phosphate buffer at pH 5. The enzymatic hydrolysis was performed using 50 mg of an AR-2000 commercial preparation with glycosidase side activity (DSM Oenology, The Netherlands) and with incubation at 40 °C for 24 h. This extract was placed into a 20-mL glass headspace sampling vial containing an equal volume of deionized water, 2 g of sodium chloride and 1.55 mg/L of 1-heptanol solution as internal standard (200 µL).

For headspace solid-phase microextraction (HS-SPME), a 50/30 µm divinylbenzene-carboxen-polydimethylsiloxane (DVB/CAR/PDMS) fiber (Supelco, Bellefonte, PA, USA) was conditioned following the manufacturer’s recommendations and exposed to the headspace of the capped vial for 20 min at 40 °C^73^. The thermal desorption of analytes was carried out at 250 °C for 5 min.

An Agilent 7890 C gas chromatograph (Santa Clara, CA, USA) coupled to an Agilent 5975 mass selective detector was used for identification and quantification purposes. The instrumental conditions were those described by Sánchez-Palomo *et al*.^73^ and slightly modified by Rolle *et al*.^71^. A DB-WAXETR capillary column (30 m × 0.25 mm, 0.25 µm; J&W Scientific Inc., Folsom, CA, USA) was used. Volatile compounds were identified using pure standards when available and/or the NIST database (http://webbook.nist.gov/chemistry/). Quantitative determinations (µg/L) were performed using the external standard calibration method with the exception of ho-trienol, for which only semi-quantitative determinations were reported. Pure standards of volatile compounds were purchased from Sigma-Aldrich (Milan, Italy) and standard solutions were prepared in 10% v/v ethanol.

### qRT-PCR analysis

For each collection points at 0, 5, 10, 15, 20 and 30% of weight loss, three biological replicates of 50 berries were randomly selected and immediately frozen in liquid nitrogen. Samples were stored at −80 °C until molecular analyses. Total RNA was extracted from deseeded berries using the Spectrum^TM^ Plant Total RNA extraction kit (Sigma-Aldrich) starting from 100 mg of material, and RNA quantity was checked using a NanoDrop 1000 spectrophotometer (Thermo Fisher Scientific, USA). Total RNA was treated with DNase I (Invitrogen, Thermo Fisher Scientific) following the manufacturer’s instructions. For each biological replicate, first-strand cDNA was synthesized starting from 500 ng of total RNA using the High Capacity cDNA Reverse Transcription kit (Applied Biosystems, Thermo Fisher Scientific) according to the manufacturer’s instructions. Reactions were carried out using specific primers (Supplementary Table S1), and Power SYBR Green PCR Master Mix (Applied Biosystems, Thermo Fisher Scientific) as reported in Gambino *et al*.^74^. Three technical replicates were run for each biological replicate, and the geometric mean of the expression ratios of two housekeeping genes (ubiquitin and actin) were used as the normalization factor. The results were calculated as expression ratios (relative quantity, RQ) to Moscato bianco berries at P0, before dehydration.

### Statistical analysis

All data were statistically analyzed using the software XLStat-Pro from Addinsoft (Paris, France). Fisher’s Least Significant Difference (LSD) test was used to analyze significant differences between AR and OZ samples (P < 0.05). Principal components analysis (PCA) was used to discriminate treatments according to terpene composition.

## Electronic supplementary material

Supplementary Table S1
